# Crack width and crack spacing in reinforced and prestressed concrete elements: Data description and acquisition

**DOI:** 10.1016/j.dib.2024.110305

**Published:** 2024-03-14

**Authors:** Anton van der Esch, Rob Wolfs, Simon Wijte

**Affiliations:** Department of the Built Environment, Eindhoven University of Technology, P.O. box 513, Eindhoven 5600 MB, the Netherlands

**Keywords:** Crack pattern, Database, Experiments, Reinforcing steel, Prestressing steel

## Abstract

Existing databases containing measurements of crack width and spacing are usually limited in size and based on isolated experimental studies. These databases are used to develop new formulas to describe crack patterns in concrete structures. A database obtained from multiple sources of experimental programmes is required to quantify the accuracy of those formulas. To this end, a database containing crack width and crack spacing measurements was created, based on 30 different experimental programs described in literature. The results of each program were described in .xlsx format and queried to a database (.csv) using Structured Query Language (SQL). The structural elements considered in the database are reinforced and prestressed ties, beams, and reinforced slabs with varying geometry, concrete and reinforcement properties. From the considered experimental programs, over twenty thousand data points were extracted using a systematic approach. The data points consist of the metadata, materials, structural element preparations, test setups and measured crack widths and spacings. The database's applied structure is robust and valuable: it can be implemented in subsequent research focussing on cracking in concrete, such as assessing existing formulas to describe the crack widths and spacings in concrete structures, or deriving new formulas, potentially improving the prediction of the remaining service life of concrete structures.

Specifications Table

**Table:** 

Subject	Civil and Structural Engineering
Specific subject area	Crack width and crack spacing in reinforced and prestressed concrete structures, subjected to axial and bending loads.
Data format	Raw
Type of data	30 Tables saved as .xlsx, each representing an experimental program (EP). Each table is indicated as EPID_xx, where xx indicates the number of the experimental program. The database (.csv), indicated as 00_Master_database.csv, was queried from the 30 tables using Structured Query Language (SQL).
Data collection	Numerical data presented in 30 publications could be straightforwardly implemented in the database. However, if data was presented in a graphical format, it was transformed into numerical data, filtered and then implemented in the database. A detailed description of the procedure is included in the methods section of this paper. The resulting database contains 24297 data points from 494 tested structural elements, described in the publications.
Data source location	The database contains secondary data from 30 experimental programs performed in Australia, Canada, New Zealand, the United States and various European countries. A detailed overview of the locations and used sources is presented below: • EPID_01: Structural Laboratory, Portland Cement Association, United States [1] • EPID_02: Munich, Germany [2] • EPID_03: Munich, Germany [3] • EPID_04: Munich, Germany [4] • EPID_05: Cornell University, New York, United States [5] • EPID_06: Instituut TNO voor Bouwmaterialen en Bouwconstructies, the Netherlands [6] • EPID_07: Building Research Station [7] • EPID_08: National Swedish Building Research, Sweden [8] • EPID_09: University of Leeds, Leeds, United Kingdom [9] • EPID_10: Imperial College of Science and Technology, London, United Kingdom [10] • EPID_11: Universität Stuttgart, Stuttgart, Germany [11] • EPID_12: Universität Innsbruck, Innsbruck, Austria [12] • EPID_13: University of Manitoba, Winnipeg, Canada [13] • EPID_14: University of Canterbury, Christchurch, New Zealand [14] • EPID_15: University of Manitoba, Winnipeg, Canada [15] • EPID_16: University of Manitoba, Winnipeg, Canada [16] • EPID_17: University of Michigan, Department of Civil Engineering, Ann Arbor, United States [17] • EPID_18: École Polytechnique Fédérale de Lausanne, Lausanne, Switzerland [18], [19], [20], [21], [22] • EPID_19: Construction Materials Laboratory, École Polytechnique Fédérale de Lausanne, Lausanne, Switzerland [23] • EPID_20: Indian Institute of Science, India [24] • EPID_21: University of New South Wales, Australia [25,26] • EPID_22: Structural Laboratory, Memorial University of Newfoundland, Canada [27] • EPID_23: Heavy Structure Laboratory, University of New South Wales, Sydney, Australia [28] • EPID_24: Structures Laboratory, Polytechnic University of Madrid, Madrid, Spain [29], [30], [31] • EPID_25: Research Laboratory of Innovative Building Structures, Vilnius Gediminas Technical University, Vilnius, Lithuania [32,33] • EPID_26: Structures Laboratory, Eindhoven University of Technology, Eindhoven, the Netherlands [34] • EPID_27: Structures Laboratory, Polytechnic University of Madrid, Madrid, Spain [[31], [35], [36]]
	• EPID_28: Norwegian University of Science and Technology, Trondheim, Norway [37] • EPID_29: Structures Laboratory, Eindhoven University of Technology, Eindhoven, the Netherlands [38,39] • EPID_30: Structures Laboratory, Eindhoven University of Technology, Eindhoven, the Netherlands [40]
Data accessibility	Repository name: Zenodo (https://zenodo.org/) Data identification number: 10.5281/zenodo.10649207 Direct URL to data: https://zenodo.org/records/10649207[41]

## 1 Value of the Data

• Researchers benefit from this database by implementing it in research to improve existing formulas or develop new formulas to describe crack patterns, consisting of the crack width and spacing. With these formulas, concrete structures' remaining service or design service life can be better predicted, potentially leading to better maintenance or replacement scheduling and fewer disturbances for users of those concrete structures.
• Data from the experimental programs was not further processed. This raw data can be used in other research projects related to crack width and spacing, and processed were needed.
• The number of variables in the database was kept as small as possible. Hence, each variable describes a unique aspect of the experiments. This makes the database easier to read and interpret.
• This paper presents a systematic approach dealing with data in graphical format encountered in publications and transforming it into numerical data, including quantification of the accuracy of this transformation process.

## 2 Background

Cracks can impair the durability performance of reinforced concrete structures and influence their esthetical appearance [42], [43], [44], [45], [46]. This makes an accurate description of crack patterns essential. Formulas are available to describe these patterns, for instance, by EN 1992-1-1 [47].

Around the 1950s and 60s, numerous experiments on crack patterns in reinforced and prestressed concrete structures were performed [1], [2], [3], [4], [5], [6],[48], [49], [50]. These experiments focussed on cracks caused by bending moments and axial forces. An extensive database with measured crack widths and spacings from experiments is needed to assess the accuracy of the formulas.

Databases typically contain less than one thousand data points, since it was outside the scope of those studies [51], [52], [53] to create extensive databases or compare numerous formulas. Recently, a categorisation of formulas and a clear database structure has been suggested [54,55]. Considering this categorisation, a new database with various geometric, material and loading properties and detailed data on the crack patterns observed in the experiments was created to assess the accuracy of crack width and spacing formulas.

This paper describes the database containing data from experiments on crack patterns in reinforced and prestressed concrete structural elements subjected to axial and bending loads.

## 3 Data Description

The database contains data obtained from 30 publications and is in the repository uploaded as 00_Master_database.csv. Each program, indicated as EPID_xx and uploaded as a .xlsx file, describes experimental programs where crack widths, w, and crack spacings, $s_{r}$, were measured. The database can be considered as a three-level structure, since each level describes the previous level in more detail. Each level of an experimental program is included in a separate tab in a .xlsx file. Further explanation can be found in the Readme and the Example file in the repository [41].

Level 1 introduces the metadata and information about the publications reporting the experimental programs used in the database. Level 2 represents the different structural elements like beams or slabs used in each experiment. Data about the geometry, the test setup, preparations of the structural elements, and the reinforcement and the concrete properties, determined with material specimens like cubes, cylinders or prisms, is also included. Finally, level 3 quantifies the load on each tested structural element and presents data on the measured crack width and spacing.

**Fig. 1:**
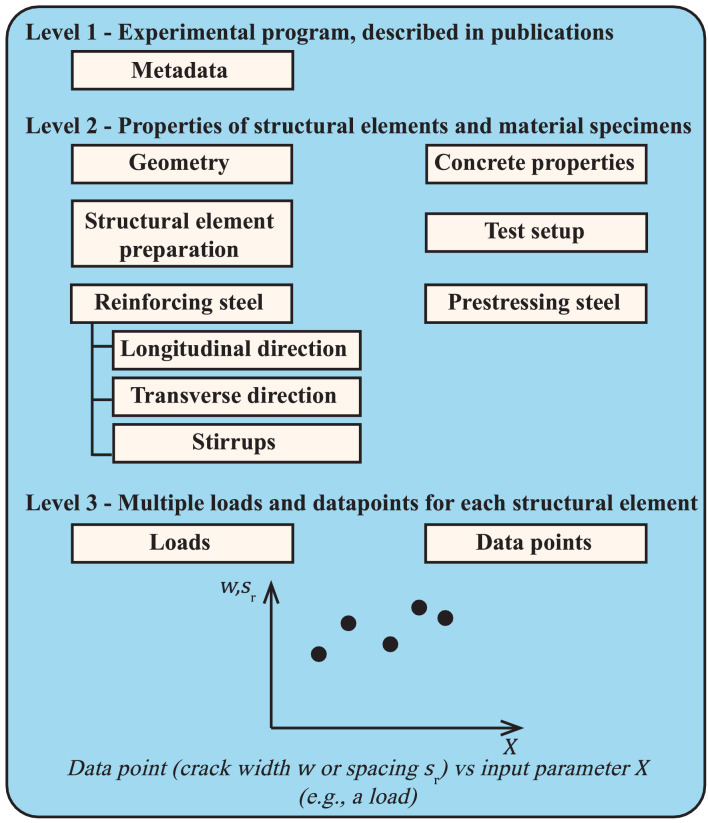
Main structure of the database, consisting of three levels, level 1: metadata describing the experimental programs, level 2: properties of structural elements and material specimen, level 3: data points, the crack width w and spacing $s_{r}$, as a function of input variables Xreported in the database.

The database structure is visualised in Fig. 1 and described in detail in the following paragraphs. The most important variables described in this paper are written in bold. These variables are necessary for describing crack widths or spacings. Furthermore, the variables described at levels 2 or 3 are partially based on the author's previous publication [54] and considered in the region of constant force.

### 3.1 Level 1 – Experimental program

The following variables characterise the information and metadata of an experimental program:

• ***epid*:** the unique identifier of the experimental program.
• *DOI*: digital object identifier of the affiliated publication.
• ***title*:** title of the publication.
• ***year*:** year of publication the publication.
• *journal*: name of the journal in which the experimental program has been published. The variable *journal* is not applicable if the experimental program has been published as a technical report or thesis.
• *country*: country of research location.
• *institution*: name of the institution where the experimental program was carried out.
• *laboratory*: name of the laboratory.
• ***authors*:** authors of the publication.

### 3.2 Level 2 – Properties of structural elements and material specimens

**Fig. 2:**
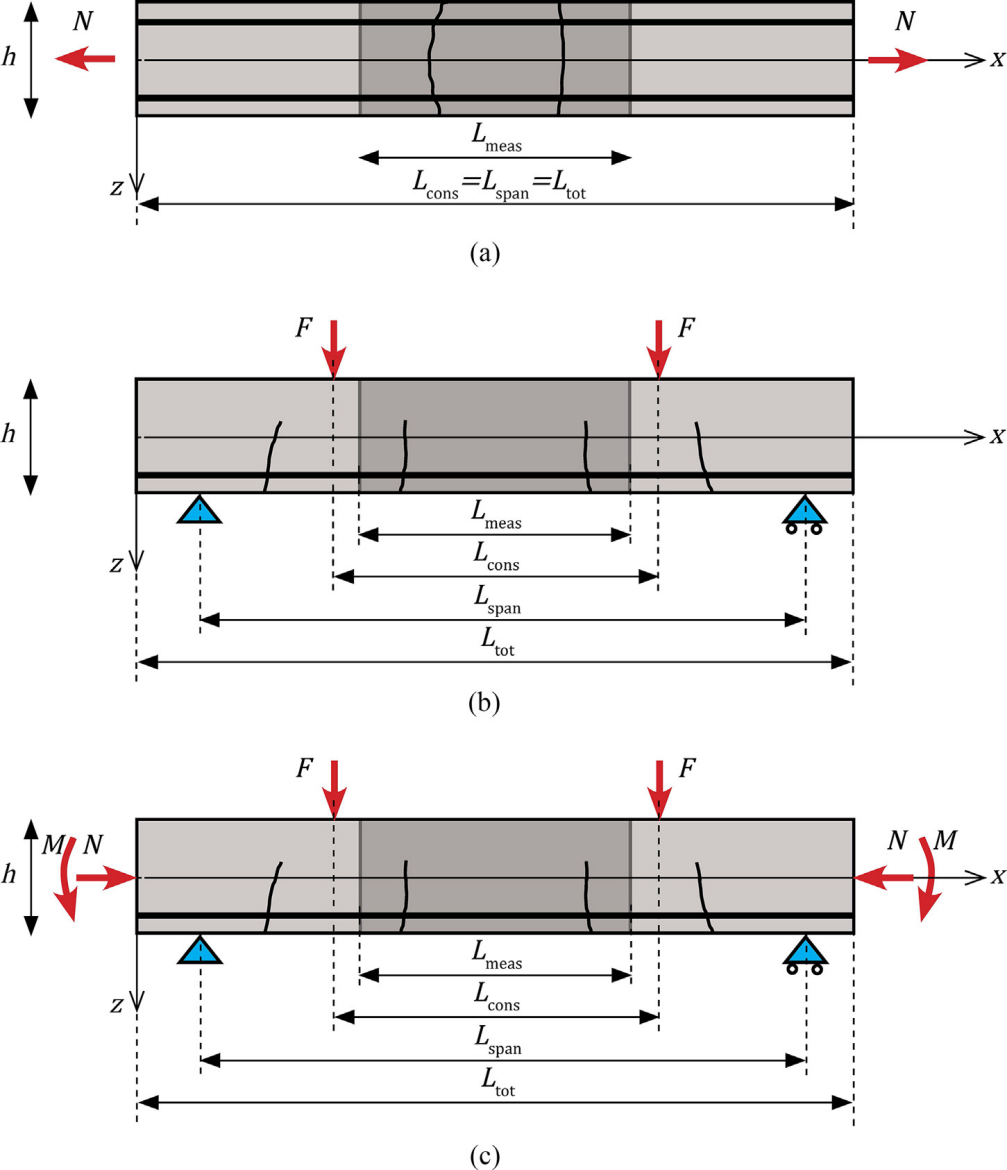
Configuration of test setups in the reported experimental programs. (a) Tie. (b) Beam in a 4-point bending test. (c) Beam loaded by bending moments and axial loads, such as a prestressed element loaded in a 4-point bending test. A constant bending moment acts in the region between the two point loads F in b) and c), designated as $L_{\text{cons}}$.

In the selected experimental programs, structural elements identified with *elid* have been loaded in multiple ways: by an axial load N (Fig. 2a), a force F in a 4-point bending test (Fig. 2b), or by a combination of axial loads and bending moments (Fig. 2c). The bending moments were introduced by F, or by a prestressing force N (Fig. 2c). The loads N and F result from force-controlled or displacement-controlled tests, or represent a prestressing load N acting on the elements.

#### 3.2.1 Geometry

The geometry of each tested structural element is described by the longitudinal and cross-sectional dimensions:

• $L_{\text{tot}}$: total length [mm].
• $L_{\mathrm{span}}$: length [mm] of span.
• $L_{\mathrm{cons}}$**:** length [mm] of the zone represented by constant axial loads or bending moments for a given load.
• $L_{\mathrm{meas}}$**:** length [mm] of the zone where the measurements were performed in the experiments.
• h**:** total height [mm] of the cross-section.
• b(z): width [mm] of the cross-section as a function of the vertical position z.

**Fig. 3:**
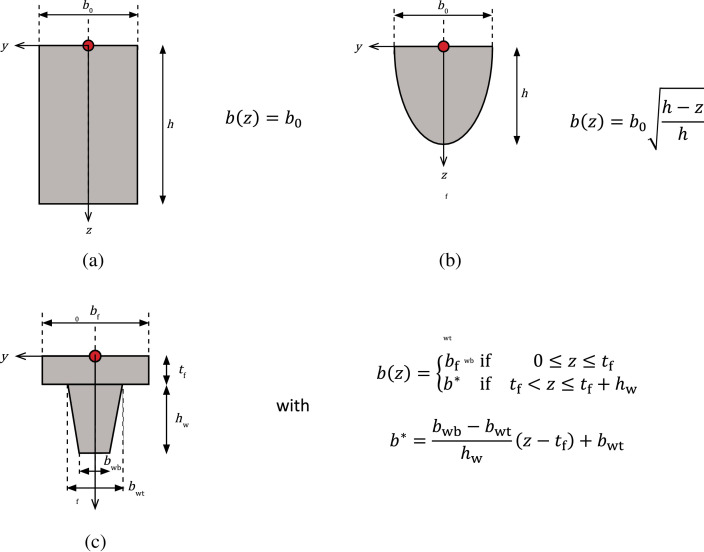
Examples of formulas for various cross-sections. The origin of the coordinate system, indicated by the red dot, is located at the most compressed fibre of the cross-section, on the vertical axis of symmetry.

The use of b(z) allows for incorporating arbitrary cross-sections with symmetry along the vertical (z) axis. Examples of cross-sections and the corresponding description of b(z) are presented in Fig. 3.

#### 3.2.2 Concrete properties

The mixture is described in accordance with EN 197-1 [56] and EN 206 [57], which helps to assess the concrete strength development during the test and can be used to estimate the shrinkage. The mixture is represented by the following variables:

• *CEM*: cement type, represented as 1 = CEMI, 2 = CEMII, 3 = CEMIII, 4 = CEMIV, 5 = CEMV.
• *class*_cem_: cement class of early strength, denoted with 1 = S (low), 2 = N (ordinary), 3 = R (high).
• *cons*: main constituents, given by 1 = blast-furnace slag, 2 = silicafume, 3 = natural pozzolana, 4 = natural calcine, 5 = siliceous, 6 = calcareous, 7 = burnt shale, 8 = limestone.
• *clinker:* clinker content, indicated as 1 = A, 2 = B, 3 = C. The clinker content A, B or C is specified in EN 197-1 [56].
• *water/cement ratio* (w/c).
• $d_{\text{max}}$: maximum aggregate size [mm].
• *type*_agg_: aggregate type, given by 1 = natural normal-weight aggregates, 2 = heavy-weight aggregates, 3 = air-cooled blast furnace slag, 4 = course recycled aggregates, 5 = lightweight aggregates.
• *additives*: applied additives in the concrete mixture, for instance, superplasticisers.
• ρ*:* volumetric mass density [kg/m^3^] of the mixture.

The modulus of elasticity and compressive and tensile strength properties of the concrete are not based on the structural elements but were determined using material specimens. Due to the varying origins of the experiments, different procedures, dimensions and shapes of material specimens were used, like cubic or cylindrical-shaped, according to EN 12390 [58] or ACI 318-19 [59]. The characteristics of the material specimen are defined by:

• $f_{\mathrm{cm}}$**:** mean value of the measured concrete compression strength [MPa].
• $t_{f_{\text{cm}}}$: age [days] of the material specimen at testing.
• *cat*_fcm_: method of measuring $f_{\text{cm}}$, specified by the *shape, dimensions* and *treatment*:
  ○ *shape*: 1 = cylinder, 2 = cube.
  ○ *dimensions*: diameter d [mm] and height h [mm] for a cylinder, length L [mm], width d [mm] and height h [mm] for a cube.
  ○ *treatment*: 1 = capped, 2 = uncapped.
• $f_{\text{ctm}}$: measured mean value of the concrete tensile strength $f_{\text{ctm}}$ [MPa].
• $t_{f_{\text{ctm}}}$: age [days] of the material specimen at testing.
• *cat*_fctm_: measurement method of $f_{\text{ctm}}$, specified by the *shape, dimensions* and *test method*:
  ○ *shape*: 1 = cylinder, 2 = cube, 3 = prism.
  ○ *dimensions*: diameter d [mm] and height h [mm] for a cylinder, length L [mm], width d [mm] and height h [mm] for a cube or prism.
  ○ *test method*: 1 = direct tensile test, 2 = tensile splitting test, 3 = flexural tensile test.
• $E_{\text{cm}}$: measured mean value of modulus of elasticity of concrete [MPa].
• $t_{E_{\text{cm}}}$: age [days] of the material specimen at testing.
• *cat*_Ecm_: measurement method of $E_{\text{cm}}$, specified by the *shape, dimensions* and *test method*:
  ○ *shape*: 1 = cylinder, 2 = cube, 3 = prism.
  ○ *dimensions*: diameter d [mm] and height h [mm] for a cylinder, length L [mm], width d [mm] and height h [mm] for a cube or prism.
  ○ *test method*: 1 = compression test, 2 = tension test.

Since concrete properties can be determined at different ages, the variables $f_{\text{cm}}\left(t\right)$, $t_{f_{\text{cm}}}$, $f_{\text{ctm}}\left(t\right)$, $t_{f_{\text{ctm}}}$, $E_{\text{cm}}\left(t\right)$ and $t_{E_{\text{cm}}}$ can consist of multiple values.

#### 3.2.3 Structural element preparations

Preparations of the structural element, represented by curing and bond conditions, influence the cracking behaviour of the concrete. Structural elements and material specimens were subjected to the same curing conditions in the selected experimental programs. The following properties relate to a specific curing condition and are partially based on EN 13670 [60]:

• *hc*: identifies the curing conditions with an integer: 1 = keeping the formwork in place, 2 = covering the concrete surface with vapour-proof sheets, 3 = placing wet coverings on the surface, 4 = keeping the concrete surface visibly wet with suitable water, 5 = application of a curing compound, 6 = stored inside test hall, 7 = stored inside climate room or subjected to climate controlled conditions, 8 = stored outside.
• $t_{\text{hc}}$: duration [days] of a specific curing condition.
• *RH*: ambient relative humidity [%].
• *T*: temperature [°C].

The variable *pd* is used to determine the bond properties between reinforcing or prestressing steel and the concrete using the orientation of the reinforcing steel while pouring the concrete: 1 = reinforcing steel is parallel to the pouring direction, 2 = reinforcing steel is perpendicular to the pouring direction.

The bond also depends on the position of the different reinforcing layers. The position is indicated by the variables $z_{s}$ and $z_{p}$ in the sections reinforcing steel and prestressing steel, respectively.

#### 3.2.4 Reinforcing steel – longitudinal direction

**Fig. 4:**
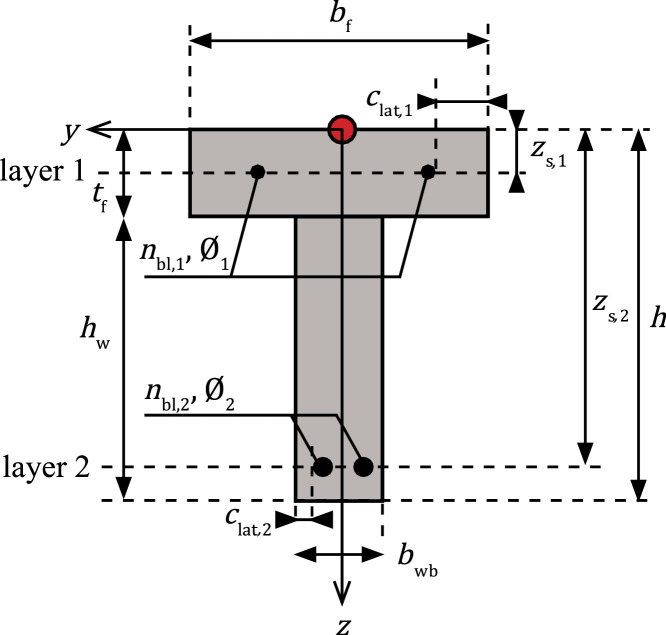
An example of a cross-section of a T-beam with reinforcing steel.

The following variables characterise the material properties of the longitudinal reinforcement (Fig. 4) in a structural element and apply to all the reinforcement layers:

• $E_{s}$: measured mean value of Young's modulus [MPa] of a tested reinforcing steel bar.
• $f_{s,r}$**:** identifies the surface characteristics of the reinforcing steel: 1 = plain, 2 = deformed.
• $f_{y}$: mean value of the yield strength [MPa].

The geometrical properties of the longitudinal reinforcing steel can be specified for each layer. Consequently, multiple layers of reinforcement can be implemented. The geometrical properties are represented by:

• ∅: nominal diameter [mm] of the applied reinforcing steel in the layer.
• $z_{s}$: the vertical distance [mm] from the origin to the layer's centre.
• $n_{\mathrm{bl}}$: number of bars in a layer.
• $c_{\text{lat}}$: lateral cover [mm] concerning the primary reinforcement's reinforcing bar, located closest to the side face of the structural element.

#### 3.2.5 Reinforcing steel – transverse direction

**Fig. 5:**
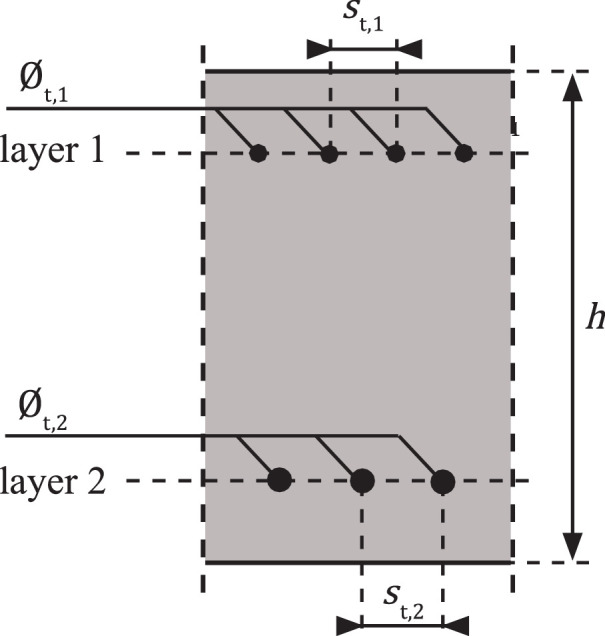
An example of a longitudinal section containing transverse reinforcement.

Reinforcing steel in the transverse direction, shown in Fig. 5, might influence the behaviour of tension and flexural cracking [60] and is therefore considered in the database. Variables determining the geometrical properties of each layer of transverse reinforcement are:

• $\emptyset_{t}$: nominal diameter [mm].
• $s_{t}$: c.t.c. (centre-to-centre) distance [mm].

It is assumed that the material properties of the reinforcing steel in the transverse direction are identical to those in the longitudinal direction.

#### 3.2.6 Reinforcing steel - stirrups

**Fig. 6:**
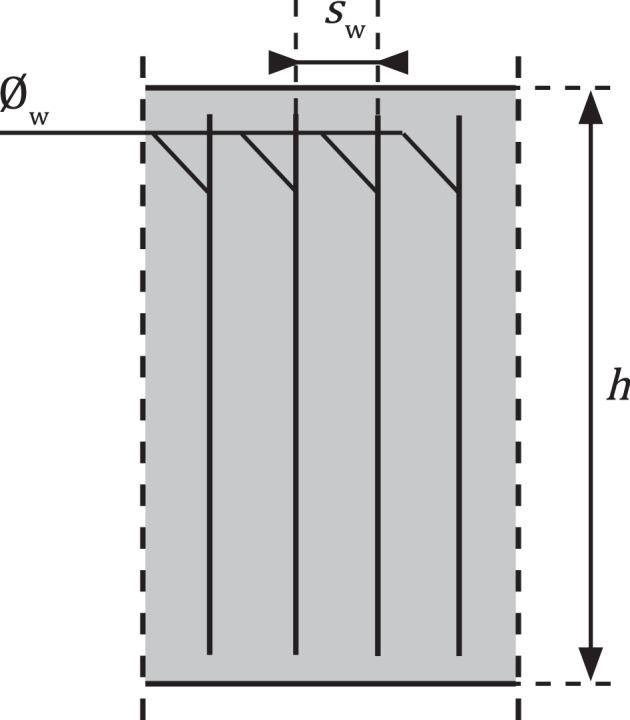
An example of a longitudinal section containing stirrups.

Besides reinforcing steel in transverse direction, steel stirrups might influence the crack pattern and are thus implemented in the database [29]. Stirrups, visualised in Fig. 6, are only included in the database if they are present in $L_{\text{cons}}$. In the database, properties of a single stirrup are included, were applicable. Considered geometrical properties are:

• $\emptyset_{w}$: nominal diameter [mm].
• $s_{w}$: c.t.c. distance [mm].

#### 3.2.7 Prestressing steel

**Fig. 7:**
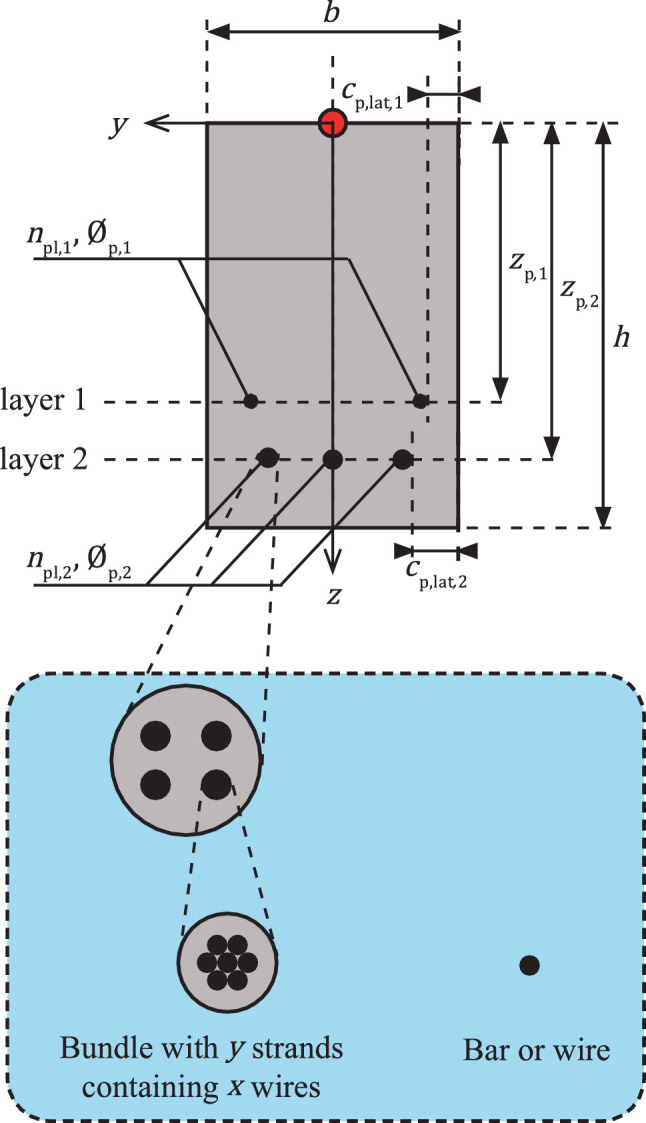
An example of a rectangular cross-section with prestressing tendons. This example contains tendons with 4 strands (y = 4) and 7 wires per strand (x = 7), hence, ySx = 4S7.

The properties of prestressing steel, visualised in Fig. 7, are described similarly to reinforcing bars in the longitudinal direction. This database considers only prestressing steel in longitudinal direction. Thus, the complete tendon profile is determined with these variables. The following variables characterise the material properties and the configuration of the tendons in an element:

• ***type*:** H = a tendon is a bar, C = a tendon is a single wire, ySx = a tendon consisting of y strands, where each strand consists of x wires. The notation of the *type* is in accordance with prEN 10138, parts 2, 3 and 4 [61], [62], [63].
• ***method***: 1 = bonded tendons with pre-tensioned steel, 2 = bonded tendons with post-tensioned steel, 3 = unbonded tendons with post-tensioned steel.
• $f_{\mathrm{pk}}$: characteristic tensile strength [MPa].
• $f_{p0.1k}$: characteristic 0.1% proof strength [MPa].
• $E_{p}$: modulus of elasticity [MPa].
• *rel*: relaxation class: 1 = class 1, 2 = class 2, 3 = class 3.
• $f_{p,r}$**:** identifies the surface characteristics of the prestressing steel: 1 = plain bar or smooth wire, 2 = ribbed bar, crimped or indented wire.

Similar to reinforcing steel, the geometric properties refer to a specific layer consisting of one or more tendons:

• $n_{\mathrm{pl}}$: number of tendons.
• $\emptyset_{p}$: nominal diameter [mm] for each tendon.
• $A_{p}$: gives the area [mm^2^] of an individual prestressing tendon.
• $z_{p}$: the vertical distance [mm] from the origin to the layer's centre.
• $c_{p,\text{lat}}$: lateral cover [mm] of a tendon, located closest to the side face of the structural element. In case *method* = 2 or 3, the lateral cover is measured from the outer side of the duct.

#### 3.2.8 Test setup

The test setup is described by the load configuration and measurement methods.

The load configuration describes how a load is applied to the tested structural element. The database considers only experimental programs where the applied loads are known. These loads, further discussed at level 3, can be represented as an axial load, a bending moment or a combination of axial and bending loads. In some experiments, the load is represented as a steel stress. This approximates the actual steel stress [64], although the approximation can be accurate if *cat*_cal_ is provided in the publication. The latter allows the calculation of the actual internal load corresponding to a particular steel stress. Publications without a known *cat*_cal_ were excluded to reduce potential erroneous stresses or loads. Structural elements loaded in pure tension are an exception, where the distribution of stresses, and thus the applied loads, is known accurately. The load configuration consists of the following variables:

• *cat*_f_*:* determines how the load in the structural element is applied, indicated by 1 = force controlled, 2 = displacement controlled.
• ${ɛ}_{\mathrm{in}}$: determines the assumed internal strain distribution over the height of the cross-section, depending on the load: 1a = constant (loaded in tension, uniaxial), 1b = constant (loaded in tension, biaxial), 2 = linear (bending), 3 = constant + linear (axial load + bending).
• ***cat*_cal_**: determines how the internal forces or stresses at a specific load level are assumed and calculated in the case of $\varepsilon_{\text{in}}=2$ or 3: 1 = linear elastic behaviour of concrete in compression, neglecting concrete in tension, 2 = nonlinear behaviour of concrete in compression, neglecting concrete in tension, 3 = nonlinear behaviour of concrete in compression, considering the contribution of concrete in tension, 4 = actual steel stresses or forces are interpolated based on the internal bending moment at failure.
• *self*: indicates if self-weight is included in the loads as indicated in the experimental programs: 1 = yes, 2 = no.

The measurement methods describe how and where the crack widths are measured:

• *type*_w_: indicates how the crack width is determined: 1 = optical strain gage, 2 = displacement transducer or extensometer, 3 = microscope, 4 = magnifying glass, 5 = digital image correlation (DIC), 6 = analysis based on resin injection in the cracks.
• ***loc*_w_**: indicates where the crack widths have been measured: 1 = at the level of reinforcement, on the side face, 2 = at the most tensioned face, 3 = at the steel-concrete interface.

### 3.3 Level 3 – Loads and data points

Each load level is indicated with a *loadid*, identifying the applied load on a structural element, the load duration and the number of load repetitions. The possible load cases are visualised in Fig. 2.

#### 3.3.1 Loads

The following variables quantify the load, except prestressing, for each load level:

• $\varepsilon_{c}\left(t_{0}\right)$: average value of the initial shrinkage strain [μs] in a tested element over the region $L_{\text{meas}}$.
• $N_{\mathrm{rep}}$: number of repetitions of the applied load for a specific load level.
• N: applied axial load [kN].
• M: applied bending moment [kNm].
• $\sigma_{s}$: steel stress [MPa] from which the actual applied external loads F and N can be calculated using *cat*_cal_.
• $t_{0}$: age [days] of structural element at first loading.
• t: duration [days] of application of the loads for *N, M*, and $\sigma_{s}$.

The prestressing loads are described with the following variables:

• ***PL***: the total applied prestressing load [kN], for each prestressing stage.
• ***cat_p_***: indicates which prestressing load *PL* is applied: 1 = total initial prestressing force before anchoring (no initial losses considered), 2 = total initial prestressing force just after anchoring (immediately occurring losses considered), 3 = total prestressing force (considering immediate and time-dependent losses).
• $t_{0,p}$: age [days] of structural element at first loading, introduced by prestressing tendons.
• $t_{p}$: duration [days] of a specific prestressing stage.

#### 3.3.2 Data points

The data points represent the results of the experiments applied for each structural element subjected to different load levels. The following variables describe the data points for a specific applied load:

• ***val***: indicates the number of cracks $N_{\text{cr}}$ within $L_{\text{meas}}$, the value of the crack width [mm] or the crack spacing [mm].
• ***valcat****:* indicates what type of *val,* which can be a crack width [mm] or spacing [mm], is quantified: 1 = the number of cracks determined, 2 = mean crack width $w_{m}$, 3 = characteristic crack width $w_{k}$, 4 = maximum crack width $w_{\text{max}}$, 5 = mean crack spacing $s_{r,m}$, 6 = maximum crack spacing $s_{r,\text{max}}$.

## 4 Experimental Design, Materials and Methods

### 4.1 Procedure of data extraction

The procedure to extract data from publications in the database started with selecting experiments with structural elements loaded by axial or bending loads.

For each experiment, it was checked whether the structural elements fulfilled the scope of the database, and all essential variables were reported. Essential variables are written in **bold** in this paper. This led to the exclusion of some described experiments, for instance, experiments where the steel stress was presented instead of the load; however, without mentioning *cat*_cal_ and a specific formula for the calculation of the steel stress [[48], [49], [50]], or the exact location of individual prestressing elements was not indicated [65,66]. For a complete overview of the excluded elements reference is made to the Readme of the database [41].

Experimental data is presented in literature in tables or as graphs. In case data was presented in graphs, first, a request was made to the authors of the specific publication to obtain the data in a numerical format. If numerical data was still unavailable, the graphs were imported into a web-based application to transform the graphs into numerical data [67]. In this application, a screenshot from the graph was imported. The screenshot was scaled, and an image rotation correction in the program was applied where necessary when the graph was obtained from a scanned publication. Then, the desired data was selected with a crosshair in the graph, and the application automatically transformed the selected point into numerical data. The transformed data mainly concerned crack widths, steel stresses or the level of externally applied loads [[1], [7], [9], [10], [18], [19], [20], [21], [24], [27], [28], [32], [34]], and incidentally, crack spacings [10].

**Fig. 8:**
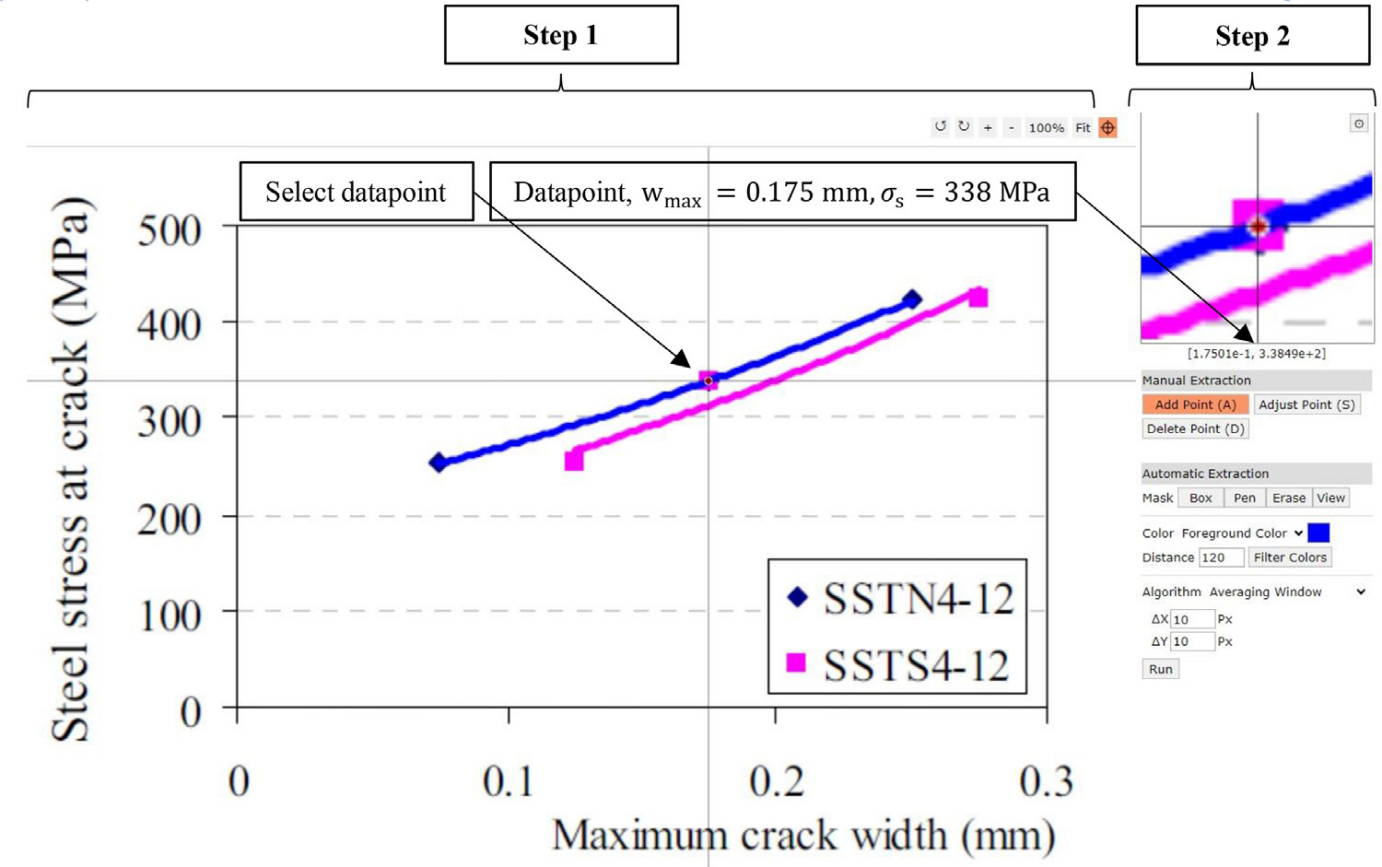
Example of the procedure to transform graphic data obtained from Wu et al. [28], into numerical data. The x-axis presents $w_{\text{max}}$, the y-axis indicates $\sigma_{s}$. Step 1) import and scale the figure in the application WebPlotDigitizer [67]. Step 2) Select a data point using the crosshair and extract the numerical data from the selected point.

**Fig. 9:**
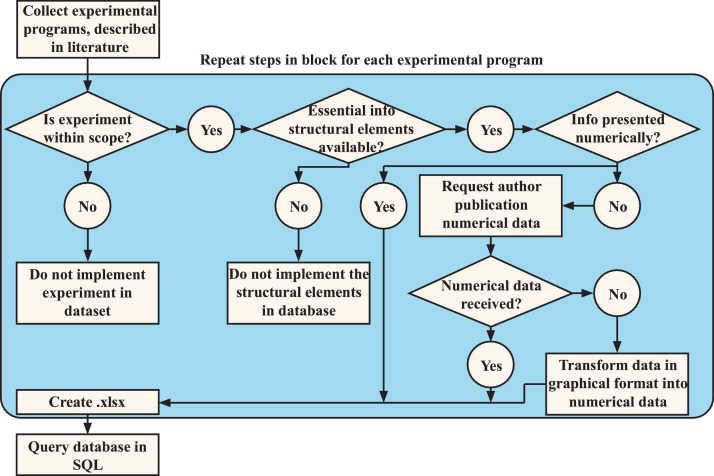
Implemented systematic approach to extract essential info from publications describing experimental programs used to create the database.

An example of transforming graphic data of crack width and steel stresses into numerical data is visualised in Fig. 8. After transforming the data into numerical data, the data was then stored in 30 tables in .xlsx format [41], where each table contains numerical data of a single experimental program. Finally, the tables were queried using SQL to obtain the database in .csv format. The complete procedure for data extraction and creation of the database is summarised in Fig. 9.

### 4.2 Determining the accuracy of data transformation

The database does not necessarily represent the variables measured during the experiments without loss of accuracy. Besides measurement errors in experiments, errors occur in transforming graphic data into numerical data. Using a systematic approach, this loss was quantified by selecting 24 graphs from three experimental programs, presenting data on crack widths and steel stresses both in graphs and a numerical format with tables [[25], [28], [49]]. From the graphs, numerical data was obtained using WebPlotDigitizer [67], see Fig. 8. Depending on availability, two to ten points per graph were selected, and the loss of accuracy due to accidental rotation of scanned graphs, limited application accuracy, and the user's accuracy was quantified with the mean ratio of the obtained numerical values. This led to an overall mean $\bar{\mu }$ of 0.994 and 1.001 and a mean Coefficient of Variance $\bar{\text{COV}}$ of 0.026 and 0.007 for the crack widths and steel stresses, respectively. Finally, besides the mean ratios, a maximum absolute error of the individual transformed graphical and numerical values was obtained: for the crack width 0.01 mm and the steel stress 1.7 MPa. These values were considered acceptable since they are order 10 and 100 magnitudes smaller (related to the measured crack width of 0.14 mm and steel stress of 156 MPa) than the measured crack widths and calculated steel stresses, respectively.

### 4.3 Implementation of the database

The described database can be implemented in subsequent research to quantify the accuracy of existing formulas that describe the crack width and spacing in concrete structures. Furthermore, it can be used to develop formulas in new standards like FprEN 1992-1-1 [68]. Moreover, it can also be implemented in parametric studies, to study the influence of specific variables on the corresponding crack width or spacing. Depending on the scope of subsequent research, each of the described variables in this paper can be filtered to obtain the data points of interest. In particular, specific types of crack widths or spacings, quantified by the variable *val*, can be obtained by filtering the variable *valcat*.

## Limitations

While the database contains an extensive amount of data points from experimental programs, it has been limited in terms of scope. For instance, the database contains measurements from programs on reinforced concrete elements or prestressed elements with straight tendon profiles, which constitute the majority of available studies. Other test or element configurations, like curved tendon profiles, are available in the literature but outside the scope of this database and, therefore, not included. In addition, some programs that are in the scope of this database could not be included due to missing data, even after contacting the author of the publication, where possible. Furthermore, elements containing reinforcing steel are represented more in the database, compared to elements with prestressing steel. Experiments with reinforcing steel are described in more detail in the literature. For some included programs, limited data was available compared to other programs. For instance, only nine tested elements were selected for the case of bi-axial tensional loading. Finally, the database contains experiments performed from the 1950s until present day. New experiments become available but are not added automatically. To prevent the database from being outdated, it can be extended with these new experiments and published as a new version.

## Ethics Statement

The authors have read and followed the ethical requirements for publication in Data in Brief and confirm that the current work does not involve human subjects, animal experiments, or any data collected from social media platforms.

## CRediT authorship contribution statement

**Anton van der Esch:** Conceptualization, Data curation, Investigation, Methodology, Resources, Validation, Writing – original draft. **Rob Wolfs:** Supervision, Validation, Writing – review & editing. **Simon Wijte:** Project administration, Supervision, Validation, Writing – review & editing.

## Data Availability

Crack width and crack spacing in reinforced and prestressed concrete elements: database (Original data) (Zenodo).
